# Autophagy machinery as exploited by viruses

**DOI:** 10.1080/27694127.2025.2464986

**Published:** 2025-03-18

**Authors:** Christian Münz, Grant R Campbell, Audrey Esclatine, Mathias Faure, Patrick Labonte, Marion Lussignol, Anthony Orvedahl, Nihal Altan-Bonnet, Ralf Bartenschlager, Rupert Beale, Mara Cirone, Lucile Espert, Jae Jung, David Leib, Fulvio Reggiori, Sumana Sanyal, Stephen A. Spector, Volker Thiel, Christophe Viret, Yu Wei, Thomas Wileman, Harald Wodrich

**Affiliations:** a Viral Immunobiology, Institute of Experimental Immunology, University of Zürich, Zürich Switzerland; b Division of Basic Biomedical Sciences, Sanford School of Medicine, University of SD, Vermillion, SD, USA; c Université Paris-Saclay, CEA, CNRS, 10 Institute for Integrative Biology of the Cell (I2BC), Gif-sur-Yvette, France; d CIRI, Centre International de Recherche en Infectiologie, Univ Lyon, Inserm, U1111, Universite Claude Bernard Lyon 1, CNRS, UMR5308, ENS de Lyon, F-69007 Lyon, France; e eINRS-Centre Armand-Frappier Santé Biotechnologie, Laval, Canada; f Department of Pediatrics, Washington University in St. Louis, St. Louis, MO, USA; g Department of Pathology and Immunology, Washington University in St. Louis, St. Louis, MO, USA; h Laboratory of Host-Pathogen Dynamics, National Heart, Lung, and Blood Institute, National Institutes of Health, Bethesda, MD, USA; i Heidelberg University, Medical Faculty Heidelberg, Department of Infectious Diseases, Molecular Virology, Heidelberg, Germany; j German Cancer Research Center (DKFZ), Division Virus-Associated Carcinogenesis, Heidelberg, Germany; k German Centre for Infection Research, Heidelberg partner site, Heidelberg, Germany; l Cell Biology of Infection Laboratory, The Francis Crick Institute, London, UK; m Division of Medicine, University College London, London, UK; n Department of Experimental Medicine, Sapienza University of Rome, Rome, Italy; o University of Montpellier, Montpellier, France; p CNRS, Institut de Recherche enInfectiologie deMontpellier (IRIM), Montpellier, France; q Department of Cancer Biology, Lerner Research Institute, Cleveland Clinic, Cleveland, OH, USA; r Guarini School of Graduate and Advanced Studies at Dartmouth, Hanover, NH, USA; s Department of Biomedicine, Aarhus University, Ole Worms Allé 4, Aarhus C, Denmark; t Sir William Dunn School of Pathology, South Parks Road, University of Oxford, Oxford, UK; u HKU-Pasteur Research Pole, School of Public Health, Li Ka Shing Faculty of Medicine, The University of Hong Kong, Hong Kong SAR, China; v Division of Infectious Diseases, Department of Pediatrics, University of California San Diego, La Jolla, CA, USA; w Rady Children’s Hospital, San Diego, CA, USA; x Institute of Virology and Immunology, Bern and Mittelhäusern, Switzerland; y Department of Infectious Diseases and Pathobiology, Vetsuisse Faculty, University of Bern, Bern, Switzerland, and Multidisciplinary Center for Infectious Diseases, University of Bern, Bern, Switzerland; z Institut Pasteur-Theravectys Joint Laboratory, Department of Virology, Institut Pasteur, Université Paris Cité, Paris, France; aa Norwich Medical School, University of East Anglia; bb Quadram Institute Bioscience, Norwich Research Park, Norfolk, UK; cc sLaboratoire de Microbiologie Fondamentale et Pathogénicité, MFP CNRS UMR, Université de Bordeaux, Bordeaux, France

**Keywords:** Endosomal damage, interferon, replication organelle, secretory autophagy, virophagy

## Abstract

Viruses adapt and modulate cellular pathways to allow their replication in host cells. The catabolic pathway of macroautophagy, for simplicity referred to as autophagy, is no exception. In this review, we discuss anti-viral functions of both autophagy and select components of the autophagy machinery, and how viruses have evaded them. Some viruses use the membrane remodeling ability of the autophagy machinery to build their replication compartments in the cytosol or efficiently egress from cells in a non-lytic fashion. Some of the autophagy machinery components and their remodeled membranes can even be found in viral particles as envelopes or single membranes around virus packages that protect them during spreading and transmission. Therefore, studies on autophagy regulation by viral infections can reveal functions of the autophagy machinery beyond lysosomal degradation of cytosolic constituents. Furthermore, they can also pinpoint molecular interactions with which the autophagy machinery can most efficiently be manipulated, and this may be relevant to develop effective disease treatments based on autophagy modulation.

## 1. Introduction on virophagy, the selective degradation of viruses by autophagy

Virus particles carry their blueprints as RNA or DNA genomes. They contain protein shells as capsids for blueprint protection. Additional proteins in the virus particles ensure host cell selection and immediate early host cell manipulation upon entry [1]. For all other aspects of their replication in host cells, viruses heavily depend on the host cell machinery which, at the same time, tries to restrict their replication, both by cell intrinsic mechanisms and communication with the host’s immune system. Therefore, successful viruses, whom we note as pathogens due to heterogenous outcomes of infections at least in immune compromised individuals, have developed strategies to evade cellular restriction mechanisms in their adaptation to at least one host species. Autophagy, a group of cellular degradation pathways for intracellular proteins, protein complexes and organelles, is no exception to this rule.

Macroautophagy, the most commonly studied of these pathways and hereafter referred to as autophagy, involves cytosolic membrane remodeling and formation of double-membrane vesicles termed autophagosomes to deliver macromolecules to lysosomal degradation [2-5]. Autophagosome biogenesis is mediated by the so-called ATG (autophagy related) proteins, which compose a protein kinase complex, a lipid kinase complex, ATG9A-positive vesicles, a lipid transfer complex, and two ubiquitin-like/Ubl conjugation systems [6]. The protein kinase complex consists of ULK1 (unc-51 like autophagy activating kinase 1) or ULK2, ATG13, RB1CC1/FIP200 (RB1 inducible coiled-coil 1) and ATG101. It is activated upon starvation, via inhibition of the MTOR (mechanistic target of rapamycin kinase) complex 1 (MTORC1) and activation of the AMP-activated protein kinase (AMPK) [6]. This complex then phosphorylates multiple targets [7], including the class III autophagy-specific phosphatidylinositol 3-kinase (PtdIns3K) complex I. This PtdIns3K complex I includes PIK3C3/VPS34 (phosphatidylinositol 3-kinase catalytic subunit type 3), PIK3R4/VPS15 (phosphoinositide-3-kinase regulatory subunit 4), BECN1 (beclin 1), ATG14, NRBF2 (nuclear receptor binding factor 2) and AMBRA1 (autophagy and beclin 1 regulator 1) and it is responsible for the synthesis of phosphatidylinositol-3-phosphate/PtdIns3P in the membranes of autophagosome precursors, known as phagophores, whose lipids are recruited from ATG9A-positive vesicles and an ATG2-containing lipid transfer complex that stabilizes channels with the ER. Phosphatidylinositol-3-phosphate formation leads to the recruitment of several effectors belonging to the ATG machinery, including WIPI1 (WD repeat domain, phosphoinositide interacting 1), WIPI2, WDR45B/WIPI3 (WD repeat domain 45B), WDR45/WIPI4 and ZFYVE1/DFCP1 (zinc finger FYVE-type containing 1). While WDR45/WIPI4 is important for the assembly of the ATG2 protein-WDR45/WIPI4 complexes, involved in the transfer of lipids from the endoplasmic reticulum (ER) to the phagophore for expansion, WIPI2 is crucial for the conjugation to phosphatidylethanolamine (PE) of the members of the Atg8-protein family, which contains six members in humans: MAP1LC3A/LC3A (microtubule associated protein 1 light chain 3 alpha), LC3B, LC3C, GABARAP (GABA type A receptor-associated protein), GABARAPL1 and GABARAPL2/GATE16. ATG8 proteins are first C-terminally processed by the ATG4 proteases (ATG4A to ATG4D), exposing a glycine that is covalently linked to PE or, in rare cases, to phosphatidylserine, through the action of the two Ubl conjugation systems [8]. For this purpose, ATG8 proteins are activated by ATG7, transferred to ATG3 and then finally conjugated to PE by the ATG12–ATG5-ATG16L1 complex. ATG12 is also an Ubl molecule that is activated by ATG7, transferred to ATG10 and then conjugated to ATG5 before associating with the ATG12–ATG5-ATG16L1 complex. ATG8 proteins which are located at the inner membrane of phagophores, mediate the recruitment of specific cargoes via the so-called selective autophagy receptors (SARs) that bridge phagophores to autophagy substrates via their LC3-interacting regions (LIRs) and ubiquitin-interacting motif (UIM)-like sequences. ATG8 proteins located at the outer membrane of phagophores mediate its elongation, autophagosome closure and recruit the machinery for fusion with late endosomes and/or lysosomes. They are finally recycled by ATG4-mediated cleavage from their lipid anchor. Autophagosomes fuse then with late endosomes and/or lysosomes with the help of specific RAB GTPases, tethering factors and SNARE proteins such as STX17 (syntaxin 17), YKT6 (YKT6 v-SNARE homolog), VAMP8 (vesicle associated membrane protein 8) and SNAP29 (synaptosome associated protein 29) [9,10], resulting in the degradation of their cargoes and of the inner autophagosomal membrane. Autophagy flux or autophagosome maturation refers to the completion of this pathway and to its cargo degradation. If this cargo is a pathogen, i.e. a bacterium or a virus, this selective type of autophagy is called xenophagy. In the case of viruses, the term virophagy is also used.

## 2. Anti-viral functions of autophagy

### 2.1 Restriction of viral entry by autophagy

Many viruses trigger autophagy early during infection. The same viruses often subsequently interfere with the late stages of autophagy, strongly suggesting that autophagy represents an innate and cell autonomous host defense. Early events associated with viral infection include virion attachment and internalization, and intracellular delivery of the viral genetic material and associated proteins. The foot-and-mouth disease virus/FMDV, a picornavirus, can interact with Arg-Gly-Asp (RGD)-binding integrins or with heparan sulfate to enter cells and both these virus binding partners appear to be able to activate an early autophagic response against the foot-and-mouth disease virus capsid proteins [11]. The complement regulating factor CD46 (CD46 molecule) mediates the attachment of vaccinal but not clinical strains of measles virus/MeV, a paramyxovirus, and rapidly induces autophagy by recruiting the PIK3C3-BECN1 complex via the CD46-Cyt1-GOPC pathway [12,13]. Similar to measles virus, the peste des petits ruminant’s virus/PPRV, another paramyxovirus, rapidly activates autophagy via the AKT1 (AKT serine/threonine kinase 1)-MTORC1 axis after binding to NECTIN4 (nectin cell adhesion molecule 4) [14]. The toll like receptor 2 (TLR2)-MYD88 axis can activate autophagy in response to the envelope gH/gL glycoproteins of herpes simplex virus 1/HSV-1 [15-17], and of human cytomegalovirus/HCMV, two orthoherpesviruses [18,19]. Upon infection of CD4^+^ T cells, the envelope glycoprotein Env of human immunodeficiency virus 1/HIV-1, a retrovirus, induces an early autophagy response that restricts viral production if not counteracted by the virus [20]. HIV-1 Env, expressed at the surface of infected cells, also induces autophagy in bystander uninfected CD4^+^ T cells, triggering their cell death [21,22]. In *Drosophila*, anti-viral autophagy has been observed *in vivo* upon recognition by Toll-7, the fly homolog of TLR7, of the glycoprotein of the vesicular stomatitis virus/VSV, a rhabdovirus [23]. Toll-7 is also important for *Drosophila* autophagic resistance to the infection by the Rift Valley fever virus/RVFV, a bunyavirus, but not to other viruses [24]. Remarkably, virion-containing endosomes can initiate antiviral mammalian autophagy through the endosomal protein SNX5 (sorting nexin 5), which activates the PtdIns3K complex I in cells infected with viruses, including the togaviruses Sindbis virus/SINV and chikungunya virus/CHIKV, West Nile virus/WNV, an orthoflavivirus, and a mutant autophagy-sensitive HSV-1. A curvature signature of virion-containing endosomes appears central for SNX5-mediated autophagy induction [25]. Activation of AMPK in response to hepatitis B virus/HBV-induced reactive oxygen species/ROS production stimulates autophagy initiation and HBV restriction [26]. Thus, virus entry or activation of pathogen-associated molecular pattern recognition receptors can stimulate autophagy during infection.

**Figure 1.**
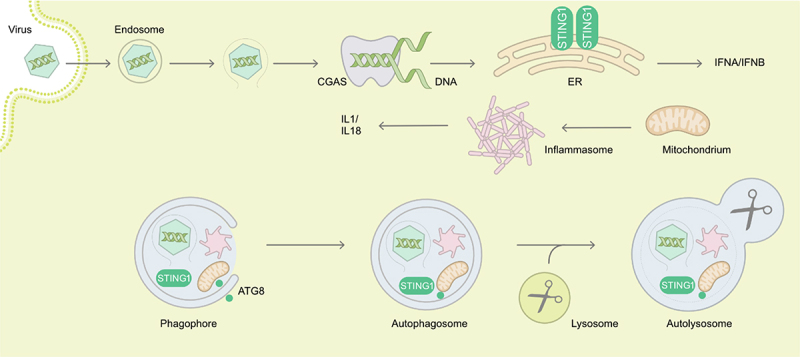
Examples of autophagy in targeting of viral entry, of sensors of viral genomes and of innate immune receptors of danger associated molecular patterns. During the cytoplasm entry by non-enveloped viruses, endosomal damage or virus capsids themselves can be recognized and targeted to degradation by autophagy. Viral DNA is detected by the CGAS-STING1 pathway, leading to type I interferon production. STING1 can also be degraded by autophagy. Damaged mitochondria activate inflammasomes triggering IL1 and IL18 production, and both are turned over by autophagy. Overall, autophagy restricts viruses during entry but at the same time dampens immune activation. ATG8, mammalian Atg8 homolog.

The detection of viral nucleic acids is another mechanism that rapidly mobilizes autophagy. Viral RNA sensing can involve TLRs [27-29], NOD-like receptors (NLRs), such as NOD2 (nucleotide binding oligomerization domain containing 2) [30,31] or, host enzymes such as EIF2AK2/PKR (eukaryotic translation initiation factor 2 alpha kinase 2) [32]. In the latter case, the autophagic degradation of HSV-1 virions is opposed by two viral proteins, Us11 and ICP34.5 [33,34]. The ICP34.5-inhibited autophagic restriction of HSV-1 appears particularly efficient in neurons [35]. Autophagy activation can also occur via the sensing of viral DNA of HSV-1 or HCMV, a mechanism that is connected with the CGAS (cyclic GMP-AMP synthase)-STING1 (stimulator of interferon response cGAMP interactor 1) pathway [18,36,37], potentially leading to viral DNA degradation (Figure 1).

Host factors can play important roles in early antiviral autophagy. The E3 Ub ligase SMURF1 (SMAD specific E3 ubiquitin protein ligase 1) is instrumental for the degradative targeting of SINV capsid by the SAR SQSTM1/p62 (sequestosome 1) [38,39]. Fanconi anemia proteins, especially FANCC (FA complementation group C) serve also as SARs for virophagy [40]. Other central regulators are some members of the RING Ub ligases of the tripartite motif-containing (TRIM) protein family, such as TRIM5/TRIM5α which opposes retrovirus infection before reverse transcription takes place [41]. TRIM5 also acts as a receptor to target the HIV-1 capsid protein p24 for autophagic degradation [41]. In Langerhans cells, the C-type lectin CD207/Langerin is central for HIV-1 internalization and TRIM5-associated degradation [42]. Other TRIM proteins are involved against either a specific or several viruses. TRIM23, which is engaged in response to infections by influenza A virus/IAV, an orthomyxovirus, HSV-1 or encephalomyocarditis virus/EMCV, a picornavirus, can promote SQSTM1/p62-driven selective autophagy via TBK1 (TANK binding kinase 1) activation and contribute to adenovirus 5, HSV-1 and SINV antiviral autophagy [43]. The targeting of capsid proteins by selective autophagy is also observed in plants as the SAR NBR1 (NBR1 autophagy cargo receptor) drives autophagic degradation of the capsid protein or virions of cauliflower mosaic virus/CaMV, a caulimovirus, in a Ub-independent manner in *Arabidopsis thaliana* [44]. Host factors can also be recruited by viruses to counter the autophagic restriction that they face during cell invasion. A prominent example is adenovirus 5 whose capsid undergoes structural changes to expose the protein VI (PVI) and ruptures the endocytic vacuole to enter the cytosol. The detection of membrane damages by particular galectins (LGALS), primarily LGALS8, along with TBK1 activation and SAR engagement triggers the formation of autophagosomes. This is antagonized by the PVI-driven recruitment of the E3 Ub ligases NEDD4 and NEDD4L, leading to delayed autophagy until viral escape from endosomal remnants is achieved [45-47] (Figure 1). Kaposi sarcoma-associated herpesvirus/KSHV also causes endosomal damage during entry, recruiting LGALS8 and CALCOCO2/NDP52 (calcium binding and coiled-coil domain 2), resulting in autophagosomal degradation of incoming virions [48]. This, however, seems to be also rapidly inhibited by KSHV after 2 h but the underlying mechanism remains unclear. HBV envelope proteins are recognized and targeted by the SAR CALCOCO2/NDP52 to the lysosome for degradation, which is independent of LGALS8 [49]. In the case of endocytosed picornaviruses, conformational changes in the capsid proteins induce small pores into the endosomal membrane for translocation of viral RNA into the cytosol. This step requires the in-situ recruitment of host PLAAT3/PLA2G16 (phospholipase A and acyltransferase 3) by damaged membranes, causing an inhibition of the LGALS8-mediated autophagic response to viral entry [50]. Thus, ubiquitination of viral components and/or glycan exposure upon endosomal damage can recruit SARs and the ATG machinery to restrict viral entry.

Non-canonical LC3B conjugation to single endosomal membranes, called LC3-associated phagocytosis (LAP), LC3-associated endocytosis/LANDO or CASM (conjugation of ATG8/LC3 to single membranes) [51-54], can also delay virus entry, as has been shown for lung epithelial cells and IAV infection [55]. In mice expressing mutant ATG16L1 that lacks the WD40 domain that is required for LC3B conjugation to single membranes, IAV fusion with the limiting membrane of endosomes was accelerated, thereby increasing infection and associated pathology. Thus, both canonical autophagy and non-canonical functions of the autophagy machinery can compromise virus entry.

Overall, these findings highlight that autophagic responses opposing viral infections can be triggered by distinct early events. Those include virus receptor binding, sensing of infection-associated membrane remodeling/perturbation (fusion, curvature, rupture) or engagement of germline-encoded receptors able to detect conserved microbial molecular patterns (envelope/capsid proteins and nucleic acids).

### 2.2 Autosis of virus infected cells

Autosis, initially described by the laboratory of Beth Levine, is a unique form of non-apoptotic, non-necrotic cell death specifically relying on ATP1A1 (ATPase Na^+^/K^+^ transporting subunit alpha 1) and autophagy [56]. Autosis was characterized by increased numbers of autophagosomes, autolysosomes, and empty vacuoles, in combination with ER dilation and fragmentation. This is followed by nuclear membrane convolution, focal ballooning of the perinuclear space, depletion of ER, reduction of autophagosome and autolysosome numbers, mitochondria swelling, and focal rupture of the plasma membrane [56]. Autotic cells are resistant to apoptosis or necrosis inhibitors, but sensitive to autophagy machinery suppression. Inhibition of autophagosome fusion with lysosome does not affect autosis, indicating that excessive autophagosome formation, rather than degradation, is probably the trigger of this process [56]. Additionally, cardiac glycosides, e.g. digoxin, which inhibit Na^+^/K^+^-ATPases, suppress autosis [56]. The interaction between BECN1 and the α subunits of Na^+^/K^+^-ATPase, i.e., ATP1A1, ATP1A2 or ATP1A3, on both intracellular membranes and at the plasma membrane is crucial, though the precise molecular mechanisms underlying autosis remain unclear.

The potential for selective autosis is a promising area of research for cancer treatments. This is illustrated by oncolytic virotherapy with myxoma virus (MYXV), a poxvirus that is endemic to rabbits and hares [57]. Preclinical studies suggest that therapies combining oncolytic viruses with tumor-specific T cells expressing chimeric antigen receptor (CAR), could enhance efficacy of this latter system, particularly, via direct injection into tumors [58]. Tumor-specific T cells rely on direct contact to induce apoptosis and pyroptosis, but this often fails to elicit strong primary responses in solid tumors or prevent resistance due to tumor antigen escape. Research by Zheng and coworkers [59] demonstrated that MYXV-infected, CAR-expressing tumor-specific T cells (CAR-TMYXV) can overcome primary resistance by delivering MYXV systemically to solid tumors. These CAR-TMYXV cells induce not only apoptosis and pyroptosis but also ATP1A1-dependent autosis through a synergy of T cell-derived IFNG/IFNγ and AKT1 signaling with the MYXV M-T5 protein-triggered SKP1 (S-phase kinase associated protein 1) and PIK3C3 signaling, significantly assisting tumor eradication.

Another example is HIV-1 infection that increases ATP1A1 expression in macrophages and memory CD4^+^ T cells, making them more susceptible to autosis when treated with autophagy-inducing peptides or nanoparticles [60,61]. The autophagy-inducing peptide Tat-beclin 1, which inhibits HIV-1 replication at low doses [62], becomes a potent inducer of autosis at higher doses or upon prolonged exposure [56,59-61,63]. This peptide selectively triggers autosis in latent HIV-1-infected primary CD4^+^ T cells and macrophages [60,61]. Similarly, the anti-apoptotic α2-helix from the death effector domain 1 of the K13 protein of KSHV, i.e., v-FLIP-α2, which impairs FAS (Fas cell surface death receptor)-dependent cytotoxic T cell killing of HIV-1-infected cells and activates HIV-1 transcription [64], also selectively induces autosis in HIV-1-infected primary resting memory CD4^+^ T cells and macrophages [60,61]. These findings highlight the therapeutic potential of autosis in treating infectious diseases and cancers, by targeting infected or malignant cells while sparing normal tissues.

### 2.3 The ATG machinery as an effector or restriction factor of interferons

Interferons (IFNs) have been recognized since the mid-twentieth century as secreted host factors that interfere with productive viral infection [65,66]. Three IFN types (I, II, III), with multiple subtypes of type I and III and a single type II, i.e., IFNG, have been identified [67]. Both the IFN system and autophagy are evolutionarily ancient mediators of the cell autonomous host defense [67-70]. As key arms of the innate immune system, autophagy and IFN coordinate an effective antiviral response. As such, viruses have evolved mechanisms to subvert the signaling and effectors of these systems to evade or co-opt the host response (see sections 3 and 4 of this review). This section will focus on how canonical autophagy and in some cases selected ATG proteins outside their role in autophagy, mediate IFN-regulated host antiviral immunity.

Autophagy is an inducible process during various physiological cues and in response to environmental stressors, including immune responses [71]. The role of IFN in the regulation of autophagy has previously been reviewed [72]. Direct and indirect evidence of type I IFN involvement in autophagy regulation exists. Type I IFN treatment induces autophagy in various human cell lines [73,74], and primary cells [75]. Consistently, IFN-inducible antiviral and IFN-regulating proteins, called IFN-stimulated genes (ISGs), are required for the autophagic control of viruses, such as EIF2AK2 during HSV-1 infection [34], RNASE1/RNAse l in EMCV and VSV infection [76], OASL (2’-5’-oligoadenylate synthetase like) in the degradation of non-structural protein 2 (nsp2) of infectious bursal disease virus/IBDV, a birnavirus, [77], and BST2/tetherin 1 (bone marrow stromal cell antigen 2) in porcine epidemic diarrhea virus/PEDV, a coronavirus [78]. Additionally, the ISG SHISA5/SCOTIN (shisa family member 5) interferes with the replication of hepatitis C virus/HCV, a hepacivirus, through the autophagic degradation of its NS5A protein [79]. In murine RAW264.7 macrophage-like cells, however, the type I IFNs IFNA/IFNα and IFNB/IFNβ did not induce autophagy [80]. Therefore, type I IFN may play a role only in specific cell types, or potentially only in the context of viral infections. Extensive evidence for IFNG induction of autophagy exists for diverse cell types, including murine RAW264.7 cells [80-83], murine embryonic fibroblasts [84], various human cell lines [77,81,82,85,86], primary macrophages [82,87,88], and primary hepatocytes [85]. Type III IFN or IFNL/IFNλ, the most recently discovered type of IFN, is also implicated in regulating autophagy. Specifically, a short form of ATG10, ATG10S, can induce degradation of various viral proteins in the presence of IFNL in human cell lines [89].

The precise factors that transduce IFN signaling to increase autophagy activity are incompletely understood. For type I IFN, MTORC1 [72] and PtdIns3K [90] signaling have been implicated. The ISG RSAD2 (radical S-adenosyl methionine domain containing 2) mediates IFNB-induced autophagy in primary human cornea-associated trabecular mesh cells [75]. In the case of IFNG, an unconventional JAK2 (Janus kinase 2)-PtdIns3K-MAPK (mitogen-activated protein kinase)-dependent, STAT1-independent, pathway may be required in murine macrophages [83], while a role for IRF1 (interferon regulatory factor 1) [85] and MTORC1 [88] has been demonstrated in Huh7 hepatocytes and human macrophages, respectively. IFNG-induced autophagy may also be countered by other cytokines that induce STAT6 (signal transducer and activator of transcription 6) [87]. As autophagy regulates many facets of the antiviral sensing and IFN response, and viruses extensively target autophagy to suppress the IFN response, selective manipulation of these mechanisms may be leveraged for therapeutic induction of antiviral IFN responses via autophagy.

Beyond a regulatory role of IFN in autophagy induction, the role of the ATG machinery as an effector mechanism has also been described for diverse virus classes. As mentioned, the ISG EIF2AK2 is required for autophagic clearance of HSV-1 virions [34,91]. HSV-1 encoded neurovirulence protein ICP34.5 antagonizes BECN1 to evade antiviral autophagy and promote viral encephalitis [91]. However, the role of IFN in protection against the BECN1-binding mutant virus remains unknown. Autophagy is also activated by HSV-1 viral genomic DNA sensing by the CGAS-STING1 pathway to limit IFN production and exerts a negative feedback loop by directly targeting viral genomes, as well as CGAS and STING1 themselves to degradation [36] (Figure 1). In order to limit RNA sensing, Sendai virus/SeV and VSV infection activates CALCOCO2/NDP52-mediated selective autophagy that targets MAVS (mitochondrial antiviral signaling protein) into degradation by a mechanism involving the ISG BST2, limiting IFN signaling [92]. Similarly, autophagy of depolarized mitochondria removes endogenous danger associated molecular patterns for inflammasome activation and pro-inflammatory cytokine production, such as IL1 (interleukin 1) and IL18 [93]. Furthermore, components of inflammasomes are also directly targeted for autophagy (Figure 1). However, the relative contribution of viral genome targeting by autophagy versus inhibition by elevated IFN and pro-inflammatory cytokine production in restricting viral replication when autophagy is inhibited, has not been dissected [36]. Furthermore, in primary mouse neurons, ATG5 restricts HSV-1 replication via a type I IFN-independent mechanism [35]. Therefore, the role of IFN in regulating HSV-1 may be cell type-specific, and redundant IFN-mediated mechanisms may exist to control HSV-1.

A role for the ATG machinery in mediating IFNG-dependent restriction of murine norovirus (MNV), a calicivirus, in a non-canonical manner has been extensively studied. Initially, it was shown that type I IFN and IFNG exhibit non-redundant activity in limiting MNV, and IRF1 is required for cell intrinsic effects of IFNG [94]. Subsequently, a role for the Ubl conjugating machineries, in particular ATG5, ATG7, and ATG16L1, were demonstrated as the mediators of IFNG activity, while LC3B, ATG4B, and lysosomal fusion mediator RAB7A were dispensable [95]. *In vivo*, mice with myeloid-specific deletion of *ATG5* infected with an acute strain of MNV exhibit increased susceptibility in the absence of type I IFN signaling, further underscoring both the non-redundant roles of these IFNs and the specificity of autophagy for IFNG [95]. The Ubl conjugation machinery required for MNV restriction also includes ATG3, and redundancy in the ATG8 homologs, while ATG14, ULK1 and ULK2 are dispensable [96]. In addition, the ISGs IGTP/IRGM3 (interferon gamma induced GTPase) and GBP2 (guanylate binding protein 2), but not IRGM1, GBP1, GBP3, GBP5 or GBP7, are required for IFNG activity, and are recruited to the MNV replication complexes to dismantle them [96]. Of note, murine GBP2 is orthologous to human GBP1.

A more complete list of ATG components required in IFNG-mediated restriction of viruses was elucidated recently. Using a candidate approach and CRISPR-Cas9-based screening of an autophagy-targeted library in the BV2 microglial cell line, ATG9A, WIPI2 and the ATG8 protein GABARAPL2/GATE16, but not LC3A, LC3B, GABARAP, or GABARAPL1, were identified as effectors of IFNG inhibition of MNV infection [97]. BECN1, ATG14, and UVRAG were dispensable [97]. Studying the role of human GBPs in HeLa cells treated with IFNG, the ATG16L1 interacting protein CAPRIN1 (cell cycle associated protein 1) was shown to mediate restriction of MNV in conjunction with WIPI2 and GBP1 [98]. IFNG also induces chicken GBP1 to restrict infectious bronchiolitis virus/IBV replication and to degrade nucleocapsid protein [89]. This activity is not pan-viral, as ECMV, murine hepatitis virus/MHV, and West Nile virus were equally well-controlled by IFNG in cells with or without ATG5 [95]. Collectively, these findings support a selective role for the Ubl conjugation machineries, together with the GBP1 system, in restricting different viruses in multiple species, without other ATG components.

The previous two decades have revealed multiple mechanisms for the regulation of both autophagy by IFNs and of viral infections by autophagy. As the evidence for IFN-regulation of viruses derives primarily from *in vitro* studies, the *in vivo* physiological relevance remains to be explored in many cases. Along these lines, the relative role of cell-autonomous IFN responses, for example type I IFN production, or IFN from immune cells, for example IFNG, in antiviral control remain to be determined. Moreover, the precise molecular mechanisms behind autophagy machinery-mediated control of viral replication, beyond degradation of virions and viral components, remains incompletely understood. Finally, the relative contribution of restricting viral infections via direct viral component degradation versus regulation of anti-viral cytokines remains poorly defined. Answers to these open questions will be important to translate our basic understanding into antiviral therapeutics.

## 3. Inhibition of the ATG machinery by viruses to prevent virophagy

Viruses benefit from inhibiting autophagy. The most obvious reason being to prevent their degradation since autophagy has been described as an antiviral mechanism. More generally, inhibition of autophagy enables viruses to create a favorable environment for their replication. This is also underscored by the fact that mutations in *ATG* genes have been associated with disease severity in patients infected by poliovirus, a picornavirus, herpes simplex virus 2/HSV-2, varicella zoster virus/VZV, another orthoherpesvirus, and possibly severe acute respiratory syndrome coronavirus 2/SARS-CoV-2 [99-102].

Many viral proteins are capable of interfering with the autophagy machinery. One of the first anti-autophagy viral protein to be identified was ICP34.5 encoded by HSV-1 [32]. ICP34.5 is a key protein for successful HSV-1 propagation in the central nervous system and multitasks in counteracting the host immune responses. Among those functions, ICP34.5 can interact with BECN1 to inhibit autophagy initiation [91]. Interestingly, the role of autophagy on the restriction of HSV-1 multiplication appears to be different depending on the cell type. The replication of HSV-1 is not modified in *ATG5* deficient fibroblasts while ICP34.5 capacity to bind BECN1 is critical for neurovirulence [35,91]. HSV-1 encodes at least two other proteins that can inhibit autophagy initiation through different mechanisms: Us3, which directly phosphorylates BECN1, and Us11, which inhibits the PKR-EIF2A pathway to prevent virus-induced autophagy [33,103].

Targeting BECN1 is one of the main strategies developed by viruses to evade autophagy and several examples can be found in different viral families. Already among the *Orthoherpesviridae* family, HCMV, KSHV and murid herpesvirus 68/MHV-68, all express proteins that block BECN1 [104-106]. Additionally, HIV-1-encoded Nef protein impairs the formation of autophagosomes by interacting with BECN1 in T cells and might also inhibit the fusion of autophagosomes with lysosomes in macrophages [107,108].

Autophagy and innate immunity are closely interconnected, and autophagy can be both a modulator and an effector of antiviral immune responses. RVFV is an arbovirus from the *Phenuiviridae* family. In *Drosophila*, RVFV triggers antiviral autophagy via the Toll-7 receptor, a mechanism that seems to be conserved in mammalian cells [24]. A recent study showed that NSs, a virulence factor encoded by RVFV, can interact with ATG8 proteins and sequester them in the nucleus [109]. These interactions are detected at later stages of RVFV infection and lead to an inhibition of autophagy. Interestingly, although a virus expressing a variant of NSs unable to bind all six ATG8 orthologs, replicates less efficiently than the wild-type virus, this difference is no longer observed in IFN-deficient cells, confirming the important crosstalk between these pathways.

Activation of autophagy with drugs was shown to restrict replication of SARS-CoV-2 in different cell lines, giving hope for a potential antiviral treatment, although the interplay between autophagy and coronaviruses seems more complex [110]. Several proteins encoded by this virus seem to be able to inhibit autophagy at different steps of the pathway. For example, expression of the accessory factor ORF3a leads to an accumulation of autophagosomes by inhibiting their fusion with the lysosomes [111]. Specifically, ORF3a disrupts the interaction between the homotypic fusion and protein sorting (HOPS) tethering complex and RAB7, preventing the tethering of autophagosomes with lysosomes. Additionally, ORF7a inhibits the autophagic flux by inducing the cleavage of SNAP29 by the CASP3 (caspase 3) protease [112]. Another respiratory virus, IAV, is capable of blocking autophagosome-lysosome fusion by expression of its structural protein M2 [113,114]. Additionally, it has also been shown that M2 is able to hijack LC3B for stable IAV particle production. Finally, adenoviruses prevent autophagosome maturation via a conserved PPxY peptide motif in their capsid protein VI that recruits the ubiquitin ligase NEDD4L/NEDD4.2. Mutating the PPxY motif in turn renders adenoviruses susceptible to autophagic clearance and increases its antigen presentation [45,115].

Some of the latest publications on viruses and autophagy shed light on the modulation of selective autophagy. Viral proteins can be targeted for autophagic degradation by SARs. However, several members of the *Picornaviridae* family encode proteases that can cleave these receptors. Two recent publications on Seneca Valley virus/SVV, a picornavirus, showed that the SARs SQSTM1/p62 and OPTN (optineurin) can target the capsid protein VP1 for turnover by autophagy and OPTN is also involved in IFN signaling via the TBK1-IRF3 pathway [116,117]. SVV encodes a protease, 3Cpro, that cleaves SQSTM1/p62 and OPTN to counteract the antiviral activity of these SARs, preventing the degradation of capsid proteins and dampening IFN response. Other picornaviruses also evolved similar strategies to block selective autophagy, like coxsackievirus B3 (CVB3), which encodes two proteinases, 2A and 3C, that can cleave SQSTM1/p62, NBR1 and CALCOCO2/NDP52 [118,119]. In the case of CALCOCO2/NDP52, interestingly, the cleavage by 3Cpro allows the formation of a stable fragment that exerts proviral properties. Reticulophagy is a form of autophagy that selectively degrades the ER via specific ER-localized SARs, such as RETREG1/FAM134B, RTN3L, CCPG1 and ATL3 [120]. The NS3 protease of several orthoflaviviruses, including dengue virus/DENV and Zika virus/ZIKV, cleaves RETREG1, whereas SARS-CoV-2-encoded ORF8 sequesters FAM134B and ATL3 in SQSTM1/p62 condensates, inhibiting reticulophagy in both cases [121,122]. Inhibition of autophagy can also be a consequence of viral subversion of selective autophagy, as it has been described for a plant-infecting pathogen called Tomato bushy stunt virus/TBSV, a tombusvirus [123]. TBSV replication protein p33 diverts both ATG8 proteins and NBR1 into its replication organelles, resulting in the inhibition of autophagy in infected cells. These latest studies provide further demonstrations that manipulation of autophagy by viruses is a widely conserved mechanism.

## 4. Pro-viral functions of autophagy

### 4.1 Building viral replication compartments with the help of the autophagy machinery

**Figure 2.**
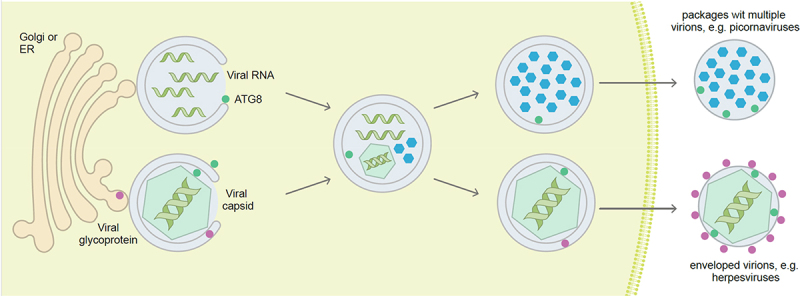
Viruses hijack autophagosomal membranes to establish or protect their replication in the cytosol, and for envelope acquisition during egress. Positive single-stranded RNA viruses often replicate in association with membrane compartments that can be assembled with the help of the ATG machinery. Once encapsulated, the viral particles can egress in virus packages within ATG8 protein-decorated membranes. Some DNA viruses also acquire their envelope from/with autophagosomal membranes and then carry parts of the ATG machinery in their virus particles. ATG8, mammalian Atg8 homolog.

Cellular stress caused by viral infection often triggers the activation of autophagy. Despite the antiviral nature of autophagy’s degradative ability, many viruses, particularly positive single-stranded RNA (+ssRNA) viruses, have evolved to resist and benefit from autophagy (Figure 2). The pro-viral nature of autophagy activation, or at least of components of the ATG machinery, is well documented for a wide variety of viruses [104,124-128]. This section focuses on how the autophagy machinery assists in the formation of virus-induced specialized organelles that harbor the replication of +ssRNA viruses.

The cytoplasmic replication of +ssRNA viruses requires membrane rearrangement to form replication factories, also called replication organelles/ROs, which concentrate and protect viral factors essential for efficient replication and transcription of the viral genomes. Pioneering ultrastructural studies of cells infected with viruses from the *Picornaviridae, Flaviviridae, Caliciviridae, Coronaviridae*, and *Arteriviridae* families by electron microscopy have revealed the cytoplasmic accumulation of double-membrane vesicles (DMVs) [129-134]. Because autophagosomes are also characterized by a double-membrane, it has been suggested that autophagy may contribute to the formation of these virus-induced DMVs [135,136], but it needs to be pointed out that the formation of DMVs might often utilize only individual components of the autophagy machinery. Notably, proteins involved in autophagy and related processes have been determined to be in close proximity to the coronavirus replication complex at DMVs [137]. Lately, 3D-reconstruction of replication organelles by electron tomography analysis confirms the presence of components of viral replicases on the inner membrane of DMVs, suggesting their involvement in the replication of at least coronaviruses [138] and hepaciviruses [139]. Importantly, the involvement of ATG proteins has been linked to DMVs formation by several +ssRNA viruses [140-144].

Coronaviruses and HCV are known to induce DMV accumulation in the cytoplasm of infected cells [139,145], to benefit from autophagy activation [144,146-148], and the direct involvement of the ATG machinery in DMV formation has been suggested [142,148-151]. However, due to conflicting data, it has remained unclear which part of the ATG machinery is essential for DMV formation. Recently, Twu and colleagues identified PtdIns3K as an essential factor for SARS-CoV-2 and HCV-induced DMV formation and their RNA replication [143], which in parallel requires the viral proteins SARS-CoV-2 nsp3 and nsp4 [150,152], or HCV NS5A and NS4B [139,153]. Interestingly, *ATG5^−/−^* Huh7-Lunet cells were still able to form virus-induced DMVs, suggesting that the autophagy elongation complex is dispensable [139,150,152,153]. Similarly, the replication of human astrovirus 1/HAstV-1, an astrovirus, localized at DMVs that required PtdIns3K for their formation [141], highlighting the importance of part of the ATG machinery in initiating DMV formation during infection. For coronaviruses, nsp6 is a multiple spanning membrane protein that localizes to the ER where it cooperates with nsp3 and nsp4 to generate DMVs as the replication organelle. Interactions between nsp3 and nsp4 extrude spherical replication organelles from the ER into the cytosol, where they house the viral RNA replicase proteins and at the same time 12 copies of nsp3 and nsp4 assemble into a pore complex to export viral RNA into the cytosol [154,155]. The replication organelles remain tethered to the ER by thin membrane connectors formed by nsp6, which induced ER zippering to collapse ER cisternae, assumed to exclude host proteins that might inhibit replication but to maintain membrane connections allowing entry of lipids [156]. A three amino acid deletion in nsp6 increased both ER zippering and virulence. Interestingly, when expressed alone in cells, nsp6 induces formation of LC3B puncta independently of activation of autophagy by starvation or inhibition of mTOR [149]. Formation of LC3B puncta by nsp6 required ATG5 suggesting conjugation of LC3 to membranes by the ATG12–ATG5-ATG16L1 complex. Nsp6 induced the formation of ZFYVE1/DFCP1-positive domains of the ER enriched for phosphatidylinositol-3-phosphate leading to recruitment of WIPI2 and the ATG8 conjugation machinery with ATG12–ATG5-ATG16L1. LC3B puncta are also induced by nsp6 of IBV, MHV and SARS-CoV-2 and the equivalent nsp5-nsp7 ortholog encoded by the arterivirus porcine reproductive and respiratory syndrome virus/PRRSV [149]. Interestingly, the LC3B puncta generated by nsp6 are small compared to LC3B puncta generated by canonical autophagy because nsp6 prevents autophagosome expansion normally seen in response to starvation [157]. Nsp6 proteins remain in the ER and do not travel with autophagosome membranes to lysosomes suggesting nsp6 disrupts early events in autophagosome formation. Affinity purification of tagged nsp6 [158] and proximity biotinylation [159] has shown that nsp6 interacts with proteins involved in membrane fusion such as SIGMAR1, VAMP7, ESYT2, ATP2A2/SERCA2 and TBK1 [159]. This complex of proteins regulates the formation of hybrid phagophore assembly sites/pre-autophagosomal structures/HyPAS generated early during autophagy by fusion of endosomal membranes containing ATG16L1 with cis-Golgi membranes enriched for RB1CC1 [159]. It is likely that nsp6 disrupts hybrid phagophore assembly sites formation to limit the maturation of autophagosomes induced during coronavirus infection.

Picornaviruses such as poliovirus and CVB3 also benefit from autophagy and induce the cytoplasmic accumulation of DMVs in infected cells [135,160,161]. Noticeably, CVB3-induced DMVs seem to rely on the autophagy machinery for their generation [140]. CVB3- and poliovirus-induced DMVs, however, appear somewhat later in the replication process [160,161]. In the case of poliovirus, it has been shown that poliovirus induced DMVs originate from cis-Golgi membranes appearing during the early stages of infection as single membrane structures that transform in a complex manner into double membrane structures [160]. Thus, additional studies on picornaviral infections are required to determine the interrelation between autophagy, DMV formation, and the viral life cycle.

HBV is a DNA virus that replicates both in the nucleus and cytoplasm. HBV infection stimulates autophagy through an increase in the PIK3C3/VPS34-BECN1 complex activity induced by the viral regulatory protein HBx [162,163]. HBV core particles are associated with the phagophore in the cytoplasm, which is required for the packaging of viral pregenomic RNA [164]. The precore protein derivatives, which form secreted viral antigens, are associated with autophagosomes. These findings suggest that differential autophagy membrane-related trafficking routes are involved in the secretion of virions and viral antigens [164].

In conclusion, the pro-viral roles of autophagy in viral replication have been well-documented for several viruses. Accumulating evidence demonstrates that virus-induced DMVs are the replication organelles of many +ssRNA viruses but the precise functions of ATG proteins in their formation still remain to be better understood.

### 4.2 Viral envelope acquisition from autophagosomal membranes

Enveloped viruses are generally composed of a capsid that houses the viral genome, enclosed in a lipid bilayer decorated with virus-encoded glycoproteins. For numerous virus families, non-glycosylated matrix or tegument proteins are found on the inner side of the envelope, ensuring the link between the capsid and the envelope. This latter is acquired by budding through either the host cell plasma membrane or organelles, such as the Golgi apparatus and the ER, but recent evidence also indicates that this can occur at autophagosome membranes (Table 1). For viruses that bud at the plasma membrane, such as IAV or rabies virus/RABV, a rhabdovius, this assembly stage also enables the release of the virion from the cell. Conversely, when the envelope originates from the endomembrane system, viral particles are transported luminally to the trans-Golgi network (TGN) and from there to the plasma membrane in vesicles to exit the cell by fusion.

**Table 1.** Viruses that modulate autophagy to favor their envelopment or pseudoenvelopment and release.

Virus	Structure/ family	Envelopment mode	Autophagy inhibition mechanism /Viral proteins	ATG proteins associated with virions	References
Budding at the plasma membrane					
RSV	ssRNA(-) linear genome *Pneumoviridae*		Autophagosome accumulation Unknown mechanism for the autophagic flux inhibition Viral protein(s) involved not identified	No information	[165]
RABV	ssRNA(-) linear genome *Rhabdoviridae*	Recruitment of RABV-M at the plasma membrane Depends on ATG5 Blocked by 3MA	Autophagic flux is inhibited by RABV-P and RABV-M RABV-P interacts with BECN1 RABV-M blocks the autophagic flux	No information	[166]
LASV MOPV	Segmented ssRNA(-) linear genome *Arenaviridae*	Recruitment of LASV-Z at the plasma membrane Depends on ATG5 and ATG7 Blocked by 3MA	Autophagosome maturation is inhibited by disruption of the cytoskeleton LASV-Z matrix protein blocks the autophagic flux and the lysosomal acidification via CCT2	No information	[168,169]
IAV	ssRNA(-) linear genome *Orthomyxoviridae*	LC3 is partially localized at the plasma membrane M2-LC3 interaction is important for filamentous viral budding	Induction of CASM but not of classical autophagy M2 interacts with LC3 and induces a non-canonical lipidation of LC3 M2 blocks the autophagic flux	Absence of LC3B proteins in the virions	[113,114,171]
HBV	Partially dsDNA circular genome/ icosahedral capsid *Hepadnaviridae*	Exploits ATG machinery for its envelopment but not for its egress An additional role in the viral pregenomic RNA packaging	Decreased levels of SNAP29 and RAB7 block the autophagic flux Non-structural viral protein HBx modulates autophagy and lysosomal acidification	ATG12	[175,177] [125,164]
PIV3	ssRNA(-) linear genome/helical capsid matrix *Paramyxoviridae*	Role in the viral release rather than in the envelopment	Inhibition of the fusion between autophagosomes and lysosomes; the phosphoprotein P interacts with SNAP29 to block the autophagic flux	No information	[175]
SFTSV	Segmented ssRNA(-) linear genome *Bunyaviridae*	Autophagy role in the envelopment into autophagosome-derived structures Complete autophagy is required	Induction of complete autophagy Nucleoprotein NP disrupts the BECN1-BCL2 association and induces autophagy	PIK3C3, BECN1, ATG14, ATG7, ATG12–ATG5, ATG16L1, LC3B-II	[178]
HCMV	dsDNA genome/ icosahedral capsid/ tegument *Orthoherpesviridae*	Presence of ATG proteins and SQSTM1 in the cytoplasmic VACs SQSTM1 colocalizes with nucleocapsids in the nucleus	Autophagy is induced and then blocked later in the infection Tegument proteins TRS1 and IRS1 interact with BECN1 and block the autophagic flux	BECN1, ATG5, ATG12, LC3B-II, SQSTM1, GABARAP	[104,179,180]
EBV	dsDNA genome/ icosahedral capsid/ tegument *Orthoherpesviridae*	Exploits autophagosome-derived vesicles for its envelopment	Autophagic flux blocked by BPLF1 through deubiquitination of SQSTM1 BVRF2/BdRF1 interaction with LC3B	ATG3, ATG4C, ATG5, ATG7, ATG8 (LC3B and GABARAPL2), SARs (SQSTM1 NBR1 and TOLLIP), PIK3C3, PIK3R4, ATG14, BECN1	[182,183] [186]
VZV	dsDNA genome/ icosahedral capsid/ tegument *Orthoherpesviridae*	Exploit autophagosome-derived vesicles for its envelopment LC3 colocalizes with VZV glycoproteins in the cytoplasm	Induction of complete autophagy	LC3B, RAB11	[181,185,201]
DENV Zika virus	ssRNA(+) linear genome/ icosahedral capsid *Flaviviridae*	Processing of prM Colocalization of E with LC3 and S-receptor kinases Release of virions within autophagosome-derived vesicles Autophagosomes contain infectious DENV	Induction of complete autophagy Lipophagy might facilitate secretion of the viral progeny	ATG proteins are associated with the infectious virion-containing extracellular vesicles	[187-189] [190,191]
HCV	ssRNA(+) linear genome *Flaviviridae*	Colocalization of APOE with E2 depends on autophagy Silencing of ATG proteins decreases HCV release and infectivity	Transient inhibition of the autophagic flux via RUBCN, a negative regulator of autophagosome‐lysosome fusion	No information	[192,194] [193,195]
Picornaviruses (e.g., poliovirus, EMCV and CVB 3)	ssRNA(+) linear genome icosahedral capsid *Picornaviridae*	Non-lytic release is enhanced by autophagy stimulation. Virus packages of at least 20 virions are released within autophagosomal membranes	Poliovirus proteins 2BC and 3A proteins lead to the accumulation of autophagosomal mamembranes	LC3B-II	[135,196-198]

dsDNA, double-stranded DNA; ssRNA(+), positive-sense single-stranded RNA; ssRNA(-), negative-sense single-stranded RNA.

Numerous studies have shown that the proviral effect of autophagy can be linked to the optimization of envelope acquisition and increased extracellular virus production. In this context, viral infections usually induce autophagosome accumulation, often associated with an inhibition of the autophagic flux, in particular by matrix proteins.

The autophagy machinery has been involved in the budding of several viruses at the plasma membrane [113,114,165-169]. For example, respiratory syncytial virus/RSV, a pneumovirus, induces the accumulation of immature autophagosomal structures by blocking autophagosome-lysosome fusion, to improve its viral production [165]. Knockdown of LC3B decreases RSV propagation and interestingly, treatment with exogenous IL22 restores the autophagic flux with concomitant reduction in RSV production. Both the mechanism of inhibition and the viral protein(s) involved in autophagy regulation are still unknown. RABV also induces incomplete autophagy in mouse neuroblastoma cells to favor its extracellular viral production [166]. At least two RABV proteins could be responsible for the accumulation of autophagosomes and the inhibition of autophagosome-lysosome fusion, i.e., the phosphoprotein RABV-P through interaction with BECN1 and the matrix protein RABV-M [166,167]. RABV-M interacts with NEDD4, an E3 Ub ligase and its ubiquitination is crucial for the accumulation of autophagosomes [166,167]. Inhibition of autophagy, by ATG5 knockdown or 3-methyladenine (3MA) treatment, decreases both RABV-M recruitment at the plasma membrane and generation of virus-like particles in the supernatant, revealing a possible role of autophagy in RABV budding [166,167]. The autophagy machinery is also hijacked by two viruses belonging to the *Arenaviridae* family, the Lassa virus/LASV, a rodent virus responsible for hemorrhagic fever in humans, and the Mopeia virus/MOPV, to favor their envelopment [168]. Silencing of ATG5 has no impact on viral DNA replication but decreases the generation of LASV and MOPV infectious particles. The matrix protein of LASV, i.e., LASV-Z, is able to impede autophagosome-lysosome fusion, lysosome acidification, and lysosomal enzyme transport by disrupting the cytoskeleton via its interaction with CCT2 (chaperonin containing TCP1 subunit 2) [169]. In the same way as for RABV-M, LASV-Z is decreased in plasma membrane-derived fractions and accumulates in the Golgi apparatus, when cells are silenced for ATG5 or ATG7, or treated with 3MA [169].

The proton-selective ion channel protein M2 of IAV is responsible for the stabilization of LC3B conjugated membranes during virus replication and egress, including SQSTM1/p62 containing vesicles with intraluminal membranes that resemble autophagosomes and amphisomes [113]. In particular, GFP-LC3B binds M2 via a highly conserved LC3-interacting region in M2, and this interaction localizes GFP-LC3B in two main pools, one in vesicles of the perinuclear region and one at the plasma membrane [114]. M2-LC3B interaction is not required for viral genome replication but is important for filamentous viral particle budding and stability of resulting virions. Indeed, knockdown of ATG16L1 decreased the proportion of filamentous particle budding. A small amount of membrane conjugated LC3B is found in extracellular viral particles and LC3B interaction with M2 that is disrupted by caspase cleavage seems required for optimal infectious particle production [170], but autophagy per se does not seem to directly contribute to IAV envelopment or egress. M2 induces an autophagy-related lipidation of LC3A/B, which nonetheless depends on ATG16L1 for the recruitment of the E3-like ATG12–ATG5-ATG16L1 complex, but it is independent of RB1CC1 and WIPI2 [171]. In fact, M2 induces the conjugation of LC3 to single membranes, in particular to the plasma membrane, through CASM. The proton-selective ion channel activity of M2 is responsible for this conjugation [172,173], via ATG16L1 recruitment by the ATP6V1H/V_1_H subunit of the vacuolar-type H^+^-translocating ATPase/V-ATPase [174]. Thus, IAV infection causes accumulation of LC3B conjugated membranes that are protected from lysosome fusion and required for stable virion production.

Manipulation of the early steps of autophagy has also been widely described for viruses acquiring their envelope through the endomembrane system, such as HBV, a hepadnavirus, parainfluenza virus 3/PIV3, a paramyxovirus, or *Orthoflaviviridae* family members. Both HBV and PIV3 block autophagosome-lysosome fusion to improve virion production. HBV decreases SNAP29 and RAB7 levels and impairs lysosomal acidification via its non-structural HBx protein [125,175]. PIV3 impairs SNARE complex formation via an interaction of its phosphoprotein P with SNAP29 [176]. Inhibition of autophagy markedly inhibited the production of extracellular virions of both HBV and PIV3, indicating a role of autophagy in viral envelopment and/or release. HBV seems to exploit autophagy for its envelopment and not for its egress, since the abundance of intracellular and extracellular enveloped HBV virions was similarly impacted by autophagy inhibition [177]. Moreover, HBV envelope proteins partially colocalize and interact with LC3B in the cytoplasm [125] and HBV core particles are associated with autophagosomes and phagophores, suggesting that these membranous structures are the site for the packaging of the viral pregenomic RNA [164]. Ding and co-workers observed that incomplete autophagy helps PIV3 extracellular viral production but does not influence viral protein synthesis or intracellular viral production, suggesting a role of autophagy in PIV3 extracellular release [176]. Severe fever with thrombocytopenia syndrome virus/SFTSV, a bunyavirus, exploits autophagy for both its envelopment, in the ER-Golgi intermediate compartment/ERGIC- and Golgi-originated autophagosomes, and its release by exocytosis [178]. Unlike HBV and PIV3, SFTSV triggers complete autophagy, via the interaction of its nucleoprotein NP with BECN1. Yan and co-workers proposed that mature SFTSV particles are released by fusion of autolysosomes with the plasma membrane.

Nucleocapsids of *Herpesviridae* family members exit the nucleus by transient envelopment across the intact nuclear membranes and acquire their final envelope in the cytoplasm before being released by exocytosis. It has been reported that several orthoherpesviruses use autophagy for their final envelopment in the cytoplasm (Figure 2). HCMV completely reorganizes pre-existing membranous compartments to create a unique compartment called viral assembly compartment (VAC), where SQSTM1/p62, LC3B and other ATG proteins are recruited [179,180]. VZV and Epstein–Barr virus (EBV), two orthoherpesviruses, generate a less-organized VAC but both exploit autophagic-derived vesicles, together with TGN- and endosome-derived vesicles for their final envelopment [181-183]. Autophagosomes accumulate in the cytoplasm of EBV, KSHV and HCMV-infected cells, due to an autophagic flux inhibition [104,182,184]. In contrast, VZV needs to induce a complete autophagy process [185]. Accordingly, the lysosomal inhibitor bafilomycin A_1_ treatment significantly decreases VZV secondary envelopment [185]. Finally, the process of orthoherpesvirus envelopment looks similar to phagophore expansion (Figure 2), and autophagosomes have been proposed as potential membrane donors for this viral envelope. Confirming this involvement, several ATG proteins are found in highly purified virions. In particular, different studies reported the presence of the lipidated form of LC3B and SQSTM1/p62 in EBV and HCMV particles and the association of LC3B and the recycling endosome protein RAB11 with VZV virions [179-182,186]. Recent mass spectrometry analysis of EBV particles identified numerous ATG proteins belonging to the PtdIns3K complex I and the Ubl conjugation systems, and SARs such as SQSTM1/p62, NBR1 and TOLLIP (toll interacting protein) [186]. Interestingly, ATG proteins are only present in a fraction of EBV virions, suggesting heterogeneity of virions with respect to their composition. The presence of ATG proteins in mature virions is not exclusive to orthoherpesviruses. It has been reported that HBV virions contain ATG12 [125] and SFSTV extracellular particles copurify with several ATG proteins, such as ATG5, BECN1 and lipidated LC3B, together with ER-Golgi intermediate compartment and TGN markers [178].

Although this is not strictly speaking for the envelope acquisition, DENV maturation depends on autophagy for the processing of its envelope glycoprotein prM and its infectivity [187]. DENV and ZIKV, two orthoflaviviruses, exit host cells in two distinct ways; one as free virions and the other enclosed within membranes. Whereas no data have connected autophagy in viral particle envelopment in the cytoplasm, the role of autophagy in the viral egress in secretory vesicles has been clearly demonstrated [188,189]. Unconventional secretory autophagy but not degradative autophagy allows the release of several infectious DENV and ZIKV particles within autophagosome-derived vesicles through a process that depends on the sequence of S-receptor kinases (Srk) [188]. A similar role of autophagy was observed for DENV in dendritic cells [190]. Moreover, hydrolysis of lipid droplets by a selective autophagy known as lipophagy plays a role in the assembly and secretion of DENV [191]. Autophagy is also involved in the maturation and release of HCV [192]. Trafficking of APOE (apolipoprotein E), a cellular apolipoprotein critical for viral morphogenesis, depends on autophagy and blocking autophagic flux improves APOE association with HCV and virion infectivity [193]. APOE is delivered by autophagosomes to the HCV assembly site where it interacts with the envelope protein complex E1/E2. HCV restricts the autophagic flux by increasing the expression of RUBCN (rubicon autophagy regulator), an inhibitor of autophagosome fusion with lysosomes [194,195].

Finally, picornaviruses, like poliovirus and CVB3, are non-enveloped viruses but they can also be non-lytically released from their host cells [196]. Stimulation of autophagy enhances non-lytic poliovirus spreading. This non-lytic spread of picornaviruses was found to occur by packages of multiple viruses, at least 20, within autophagosomal membranes [197,198] (Figure 2). These viruses utilize phosphatidylserine in the outer leaflet of autophagosomal membranes, which mostly originate from the ER and Golgi apparatus, to enter host cells via scavenger receptors such as TIMD4/TIM-4 (T cell immunoglobulin and mucin domain containing 4) and MERTK (T cell immunoglobulin and mucin domain containing 4) [197]. This mechanism increases viral spreading. In addition, membranes around the virus packages protect their content from environmental harm, such as antibodies. Along these lines, vesicle cloaked viruses have even been shown to survive fecal to oral transmission [199]. Therefore, both enveloped and non-enveloped viruses hijack the autophagy machinery for release in autophagosomal membranes and efficient spreading.

## 5. Conclusions and outlook

Some of the first electron microcopy pictures of double-membrane vesicles originated from virus infected cells [134], around the time when Christian de Duve had coined the term autophagosome [200]. The information gathered in this review suggests that nearly every virus has to cope with the anti-viral activity of autophagy and many modulate this pathway for their survival. Some viruses even utilize the machinery that generates autophagosomes for building their replication compartments and/or acquiring envelopes. Both of these strategies, inhibition of autophagosome formation and blocking autophagosome fusion with lysosomes, inhibit virophagy. Thus, autophagy stimulation might overcome these viral interference mechanisms and retore autophagic flux for virion or virus component degradation. A detailed understanding of how every virus interacts with autophagy might also enable us to more specifically tilt the balance of pro-viral and anti-viral functions of autophagy during viral infections for their treatment.

## Abbreviations

ATG, autophagy related; BECN1, beclin 1; CAR, chimeric antigen receptor; CASM, conjugation of ATG8/LC3 to single membranes; CVB3, coxsackie virus B3; DENV, dengue virus; DMVs, double-membrane vesicles; dsDNA, double-stranded DNA; EBV, Epstein–Barr virus; EMCV, encephalomyocarditis virus; ER, endoplasmic reticulum; HBV, hepatitis B virus; HCMV, human cytomegalovirus; HCV, hepatitis C virus; HIV, human immunodeficiency virus; HSV, herpes simplex virus; IAV, influenza virus; IFN, interferon; ISG, interferon-stimulated gene; KSHV, Kaposi sarcoma associated herpesvirus; LASV, Lassa virus; MHV-68, murid herpesvirus 68; MNV, murine norovirus; MOPV, Mopeia virus; MYXV, myxoma virus; Nsp, non-structural protein; PE, phosphatidylethanolamine; PIV, parainfluenza virus; PRRSV, porcine reproductive and respiratory syndrome virus; PPRV, peste des petits ruminant’s virus; PtdIns3K, class 3 phosphatidylinositol 3-kinase; RABV, rabies virus; RSV, respiratory syncytial virus; RVFV, Rift Valley fever virus; SAR, selective autophagy receptor; SARS-CoV-2, severe acute respiratory syndrome coronavirus 2; SFTSV, severe fever with thrombocytopenia syndrome virus; SINV, Sindbis virus; ssRNA, single-stranded RNA; TBK1, TANK binding kinase 1; TGN, trans-Golgi network; TRIM, tripartite motif containing; Ub, ubiquitin; Ubl, ubiquitin-like; VAC, viral assembly compartment; VSV, vesicular stomatitis virus; VZV, varicella zoster virus
